# Fibroblast Growth Factor 21 Augments Autophagy and Reduces Apoptosis in Damaged Liver to Improve Tissue Regeneration in Zebrafish

**DOI:** 10.3389/fcell.2021.756743

**Published:** 2021-10-22

**Authors:** Weidong Qiang, Tianzhu Shen, Muhammad Noman, Jinnan Guo, Zhongqian Jin, Danfeng Lin, Jiaxuan Pan, Huiqiang Lu, Xiaokun Li, Fanghua Gong

**Affiliations:** ^1^College of Life Sciences, Jilin Agricultural University, Changchun, China; ^2^School of Pharmacy, Wenzhou Medical University, Wenzhou, China; ^3^Center for Drug Screening and Research, College of Geography and Environmental Engineering, Gannan Normal University, Ganzhou, China; ^4^Center for Developmental Biology of Jinggangshan University, College of Life Sciences, Jinggangshan University, Ji’an, China

**Keywords:** FGF21, damaged liver, regeneration, autophagy, zebrafish

## Abstract

Regeneration of a part of the diseased liver after surgical resection is mainly achieved by the proliferation of the remaining healthy liver cells. However, in case of extreme loss of liver cells or in the final stages of chronic liver disease, most liver cells are depleted or lose their ability to proliferate. Therefore, to foster liver regeneration, it is of great clinical and scientific significance to improve the survival and proliferation ability of residual hepatocytes. In this study, we conducted experiments on a zebrafish model of targeted ablation of liver cells to clarify the role of fibroblast growth factor 21 (FGF21). We found that FGF21 increased the regeneration area of the damaged liver and improved the survival rate of damaged liver cells by inhibiting cell apoptosis and reducing oxidative stress. Our results also showed that administration of FGF21 upregulated autophagy, and the beneficial effects of FGF21 were reversed by the well-known autophagy inhibitor chloroquine (CQ), indicating that FGF21-activated autophagy played a central role in the treatment. We further showed that the enhancement of autophagy induced by FGF21 was due to the activation of the AMPK-mTOR signaling pathway. Taken together, these data provide new evidence that FGF21 is an effective autophagy regulator that can significantly improve the survival of damaged livers.

## Introduction

Growth factors, especially fibroblast growth factor (FGF), have been reported to increase the protection and viability of the liver (Yang et al., 2013). FGF21 has shown good therapeutic and protective effects against ethanol-induced liver injury and fructose-induced fatty liver (Zhao et al., 2019). Nishimura et al. (2000) were first to isolate FGF21 from mouse embryonic tissue; they subsequently showed the regulatory function of FGF21 in the metabolism of a variety of organisms (Seo and Kim, 2012). For example, in mice on fasting or ketogenic diet, the hepatic content of FGF21 increased dramatically, which was accompanied by an increase in the level of FGF21 in the blood circulation (Badman et al., 2007). Oxidative stress caused by reactive oxygen species in the body leads to the production of lipid peroxides, which can damage the liver and accelerate liver cell apoptosis (Koek et al., 2011; Cui et al., 2021). Therefore, after the liver has been damaged, timely reduction of oxidative stress can aid liver regeneration (Lu et al., 2021). Recently, FGF21 has been proven to inhibit oxidative stress and apoptosis and promote autophagy (Yan et al., 2018; Huang et al., 2019; Zhou et al., 2019). FGF21 also corrects multiple metabolic parameters of non-alcoholic fatty liver disease *in vitro* and *in vivo* by upregulating autophagy to maintain liver cell survival and regeneration (Zhu et al., 2016). FGF21 treatment in obese mice can improve Jumonji-D3 histone-dependent autophagy and liver steatosis (Byun et al., 2020). Therefore, FGF21 can promote liver regeneration by enhancing autophagy of damaged liver cells, inhibiting oxidative stress, and inhibiting cell apoptosis.

We used transgenic zebrafish [*Tg(lfabp:Dendra2-NTR)^*cq1*^*] to evaluate the effect of FGF21 on liver regeneration. In this fish line, targeted ablation of liver cells was induced by bacterial nitroreductase (NTR) and its substrate metronidazole (MTZ). Our results showed that FGF21 treatment after liver injury could upregulate the expression of superoxide dismutase (SOD) and downregulate the expression of malondialdehyde (MDA), catalase (CAT), acute suppurative thyroiditis (AST), and alanine aminotransferase (ALT); it also effectively inhibited apoptosis of damaged liver cells, upregulated the expression of the anti-apoptotic gene *bcl2*, and downregulated the expression of apoptosis genes *bax* and *p53*; the expression of autophagy marker protein LC3B was also upregulated. In addition, using molecular biology methods, we showed that the underlying mechanism by which FGF21 improved liver regeneration was dependent on the activation of the AMPK-mTOR signaling pathway. In conclusion, our results showed that the action of FGF21 upregulated autophagy of hepatocytes and shortened the overall process of liver regeneration.

## Materials and Methods

### Experimental Materials

Fibroblast growth factor 21 standard protein was provided by Wenzhou Medical University (Wenzhou, China); metronidazole was purchased from Shenggong (Shanghai) Biotechnology Co., Ltd.; chloroquine (HY-17589A) was purchased from MCE Biotechnology Co., Ltd.; and glutathione (GSH), malondiazole aldehyde (MDA), superoxide dismutase (SOD), and catalase (CAT) detection kits were purchased from Nanjing Jiancheng Institute of Bioengineering (Nanjing, China). Bicinchoninic acid (BCA) protein assay kits were purchased from Thermo Fisher Scientific (Rockford, Illinois, United States), and the ECL Plus kit was purchased from PerkinElmer Life Sciences (Waltham, MA, United States). The TUNEL apoptosis detection kit (Alexa Fluor 640) was purchased from Yeasen Biotechnology Co., Ltd. (Shanghai, China), and qPCR reagents were purchased from Takara (Dalian, China).

The following primary antibodies were purchased: PCNA and LC3B from Abmart Inc. (Shanghai, China); Anti-p53, Anti-bcl2, Anti-bax, Anti-AMPK, Anti-p-AMPK, Anti-mTOR, Anti-p-mTOR, and Anti-SQSTM1/p62 from Affinity Biosciences (Cincinnati, OH, United States); and 4’,6-diamidino-2-phenylindole (DAPI), protein loading buffer, and β-actin from Beyotime Biotechnology (Jiangsu, China).

### Fish Breeding and Embryo Collection

The transgenic *Tg (lfabp: Dendra2-NTR)^*cq1*^* zebrafish strain was provided by the Drug Screening Center of Gannan Normal University, and the wild-type (WT) AB strain zebrafish was purchased from the China Zebrafish Resource Center. In line with Instructional Animal Care and Use Committee (IACUC), all zebrafish were raised in aerated freshwater flow tanks under a cycle of 14 h of light and 10 h of darkness at 28 ± 0.5°C. In accordance with the recommendations from Lu et al. (2013), newly hatched braised shrimp were used to raise fish. When collecting embryos, males and females were introduced into the breeding tank at a ratio of 1:1 and separated by a partition. The next morning, the partition was removed, and the females began laying embryos. The feces, debris, and dead and unfertilized eggs were discarded. The surviving embryos were washed with pure water several times and incubated at 28.5°C for 24 h. The culture medium was replaced with 1-phenyl-2-thiourea (PTU) fish juice to inhibit melanin production.

### Establishment of the Liver Injury Model and Treatment Plan

Five-day-old juvenile fish were incubated with 12 mmol/L metronidazole (He et al., 2014) in 0.2% dimethyl sulfoxide (DMSO) for 36 h and then washed three times in the culture solution. After examination under a Leica stereo fluorescence microscope, juveniles with damaged liver were selected and distributed to a 6-well plate at a density of 20 juveniles per well.

The selected juveniles were randomly divided into four groups: injury control group (MTZ, *n* = 40); post-injury FGF21 treatment group (MTZ+FGF21, *n* = 40); post-injury, FGF21–CQ co-treatment group (MTZ+FGF21+CQ, *n* = 40); and negative control group (DMSO, *n* = 40). After establishing the liver injury model, the juvenile fish were treated with 200 ng/mL of FGF21 solution (Supplementary Table 1); DMSO solution was used as negative control, and all treatment groups were administered once every 24 h; FGF21 (200 ng/mL/24 h) and CQ (5 μM/24 h) were used to treat MTZ+FGF21+CQ juveniles according to the same protocol. The embryos were incubated at 28.5°C until 48 hpf, and the reagents were replaced every 24 h. The whole process was photographed and recorded, and we counted the number of dead embryos. The experiment was repeated three times.

### Fluorescence Microscopy

Differentially treated juvenile fish were sedated with 0.15% tricaine at 24 and 48 h post administration (hpa) and maintained in 1% low-melting agarose gel. We used tweezers to place a single zebrafish in the plate so that the eyes, body parts, and the tail were at the same level. A fluorescence microscope (Leica TCS SP8, Germany) was used to visualize and capture images of the liver in each treatment group.

### Antibody Staining

Differentially treated juvenile fish were collected from each group at 0, 24, and 48 hpa, washed three times with 1 × PBS, fixed with 4% paraformaldehyde on a shaker at 4°C overnight, dehydrated by gradient methanol overnight, made transparent with acetone, and washed with 3% PT (0.1% Triton X-100+PBS). The treated juveniles were then blocked with PBTN (0.1% Triton X-100 + 5 mg/mL bovine serum albumin + PBS + Tween 20) on a shaker at 4°C for 2 h and incubated with primary antibodies (anti-PCNA and anti-LC3B) on a shaker at 4°C overnight. After washing with 3% PT, the samples were counterstained with DAPI for 30 min, and the cells were visualized under a laser scanning confocal microscope (Leica TCS SP8, Germany).

### TUNEL Staining

Following the manufacturer’s instructions, TUNEL staining was used to assess DNA damage. In short, the embryos fixed with 4% paraformaldehyde were digested by proteinase K (20 μg/mL) and stained with the in situ cell death detection kit at 37°C for 2 h. After washing these embryos three times with PBTN, they were counterstained with DAPI for 30 min. TUNEL positive cells were visualized using a fluorescence microscope (Leica TCS SP8, Germany).

### Western Blotting

The livers of 40 larvae were collected from each group at 0, 24, and 48 hpa. The samples were then homogenized in radio immunoprecipitation assay (RIPA) lysis buffer (Santa Cruz Biotechnology Co., Ltd., Dallas, TX, United States) to produce a lysate of total tissue protein. The total tissue protein was determined by the BCA assay method to ensure that the sample amount per channel was 30 μg. The lysate was diluted at a ratio of 1:5 with the protein loading buffer and boiled at 100°C for 10 min. The protein extract was separated by sodium dodecyl sulfate–polyacrylamide gel electrophoresis (SDS-PAGE) (12% gel) and transferred to a polyvinylidene fluoride membrane (Immobilon P, Millipore, United States). The membrane was incubated with 5% milk in triethanolamine buffered saline-tween (TBST) on a shaker at room temperature for 2 h; the primary antibody was added and incubated at 4°C overnight, followed by incubation with horseradish peroxidase (HRP)-conjugated secondary antibody on a shaker at room temperature for 2 h. The following primary antibodies (1:2000) were used: anti-p53, anti-bcl2, anti-bax, anti-MAPK, anti-p-MAPK, anti-mTOR, anti-p-mTOR, anti-LC3B, anti-P62, and anti-β-actin. Secondary antibody (goat anti-rabbit IgG-HRP 1:2000) was obtained from Bioss (Beijing, China). An enhanced high-sig ECL western blotting substrate was used, and imaging was performed by a ChemiDoc XRS Plus luminescence image analyzer (Bio-Rad). The Quantity one software (Bio-Rad, Hercules, CA, United States) was used to analyze the gray intensity of each band.

### Real-Time Fluorescence Quantification PCR

Using TRIzol (Takara, Tokyo, Japan), the total RNA was extracted from the livers of 40 larvae. Next, cDNA was synthesized from 1 μg of total RNA of each sample using PrimeScript^TM^ RT reagent kit (Takara, Tokyo, Japan). Quantitative real-time PCR (qRT-PCR) was performed on a real-time PCR system using SYBR^®^ Premix Ex TaqTM. Supplementary Table 2 lists the zebrafish primer sequences that were used for qPCR analysis in this study. The relative gene expression levels were calculated with β-actin as a housekeeping gene. The experiments were performed in triplicates.

### Oxidative Stress Determination

The juvenile fish were collected at 24 and 48 hpa, and the total protein of 40 livers were evaluated using the Kamas Brilliant Blue Analysis Kit. The absorbance was recorded using a multifunctional spectrophotometer (PerkinElmer Victor Nivo, United States). The contents of MDA, CAT, and SOD were determined using the corresponding kits. Subsequently, ALT and AST enzyme activity and contents were determined in accordance with the instructions on the kit. The activity of AST and ALT in the tissue (U/g, in terms of protein amount) was determined as AST and ALT activity of the homogenate obtained from the standard curve (U/L) divided by the protein of the homogenate to be tested (g/L). The experiment was repeated three times.

### Statistical Analysis

The data were analyzed statistically using GraphPad Prism software and presented as mean ± standard deviation (SD). One-way analysis of variance (ANOVA) was used for multiple-group comparisons. *P* < 0.05 was considered statistically significant.

## Results

### Fibroblast Growth Factor 21 Improves Liver Regeneration After Induced Injury

**FIGURE 1:**
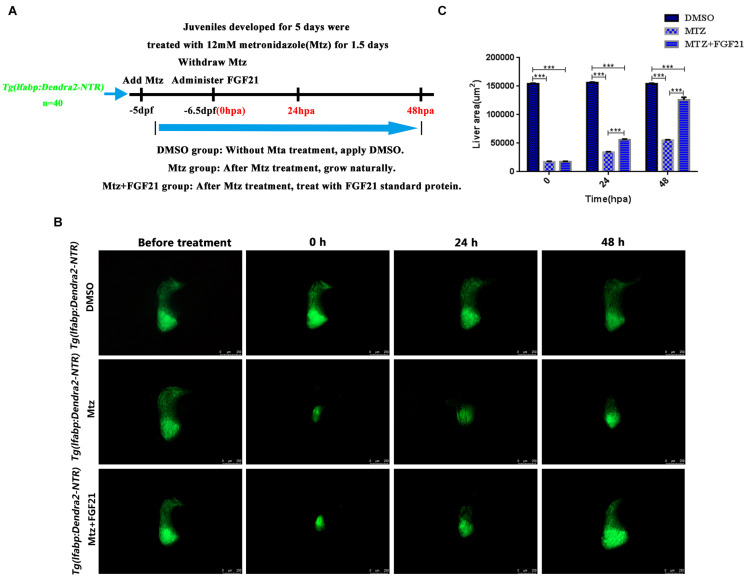
Fibroblast growth factor 21 improves liver regeneration after induced injury. After establishing the liver targeted-ablation model, FGF21 was administered and the treatment effect was evaluated. **(A)** The overall experimental process of FGF21 therapy for targeted liver ablation. **(B)** Liver regeneration at several time points after FGF21 treatment of the targeted ablated liver. **(C)** The histogram shows the quantification of the regenerated liver area assessed by ImageJ. DMSO group: without Mtz treatment, DMSO was applied. Mtz group: after Mtz treatment, the growth is natural Mtz+FGF21 group: after Mtz treatment, they were treated with FGF21 standard protein. Significance: ***p< 0.001 vs. DMSO group. Data are expressed as mean± standard error of mean (SEM) (*n* = 40 per group, repeat 3 times for each test). Scale bar = 250 μm.

The *Tg (lfabp: Dendra2-NTR) ^*cq1*^* transgenic line was produced by the fusion of the fluorescent protein Dendra2 and NTR and driven by the hepatocyte-specific promoter lfabp. After the incubation of five dpf (days postfertilization) of *Tg (lfabp: Dendra2-NTR)^*cq1*^* larvae with 12-mmol/L Mtz for 36 h, hepatocytes showed ablation (Figures 1A,B). Subsequently, Mtz was removed, and zebrafish with 25% of the normal liver size were selected for administration of FGF21 and FGF21+CQ. In *Tg (lfabp: Dendra2-NTR)^*cq1*^* transgenic larvae treated with Mtz and Mtz+FGF21, regeneration of liver cells was observed (Figure 1B); the liver regeneration rate in the group treated with Mtz+FGF21 was significantly higher than that in the Mtz group (Figures 1B,C). Qualitatively, the missing part of the liver in the control group was still large at 24 hpa after the removal of Mtz; however, after FGF21 treatment, liver regeneration was already prominent (Figure 1B). The liver regenerated to 80% of its normal size at 48 hpa (Figure 1C) and regenerated completely in the next 24–48 h. In summary, these results indicate that FGF21 plays a key role in liver regeneration, and exogenous administration of FGF21 can significantly improve liver regeneration.

### Fibroblast Growth Factor 21 Inhibits Liver Cell Apoptosis After Injury

**FIGURE 2:**
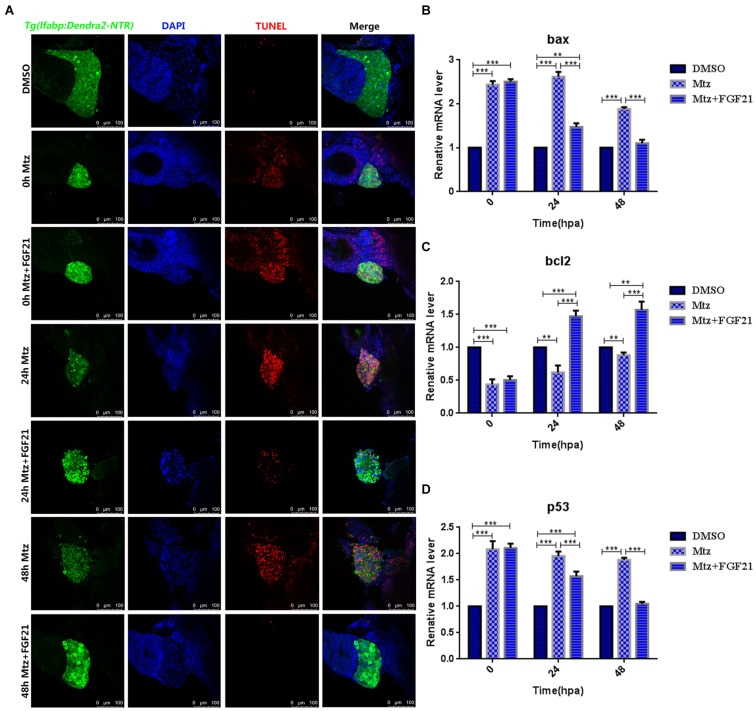
Fibroblast growth factor 21 inhibits liver cell apoptosis after injury. **(A)** TUNEL assay performed to assess the level of DNA damage in the damaged liver (nuclei, blue; damaged DNA, red; and liver cells, green). **(B)** RT-qPCR detection of *bax* mRNA of three groups at 0, 24, and 48 hpa. **(C)** RT-qPCR detection of *bcl2* mRNA of three groups at 0, 24, and 48 hpa. **(D)** RT-qPCR detection of *p53* mRNA of three groups at 0, 24, and 48 hpa. DMSO group: without Mtz treatment, DMSO was applied. Mtz group: after Mtz treatment, grow naturally. Mtz+FGF21 group: after Mtz treatment, treated with FGF21 standard protein. Significance: **p< 0.01, and ***p< 0.001 vs. DMSO group. Data are expressed as mean ± SEM (*n* = 40 per group, repeat 3 times for each test). Scale bar = 100 μm.

In order to verify that FGF21 plays a key role in liver regeneration, we examined the rate of liver cells’ apoptosis after injury. Compared with the Mtz group, apoptosis of liver cells was significantly inhibited in the group treated with Mtz+FGF21 (Figure 2A). At 24 hpa after removal of Mtz, there were still more cells undergoing apoptosis in the Mtz group; however, after FGF21 treatment, cell apoptosis was significantly inhibited (Figure 2A and Supplementary Figure 1). In addition, proapoptotic genes including *bax* and *p53* were significantly downregulated after FGF21 treatment (Figures 2B,D), while the antiapoptotic gene *bcl2* was upregulated (Figure 2C), at the same time, we tested the protein expression levels of apoptosis-related factors p53, bcl2, and bax, and the results were consistent with their gene expression (Supplementary Figure 2). Therefore, compared with the Mtz group, FGF21 treatment can effectively inhibit liver cell apoptosis after injury.

### Fibroblast Growth Factor 21 Reduces Oxidative Stress After Liver Injury

**FIGURE 3:**
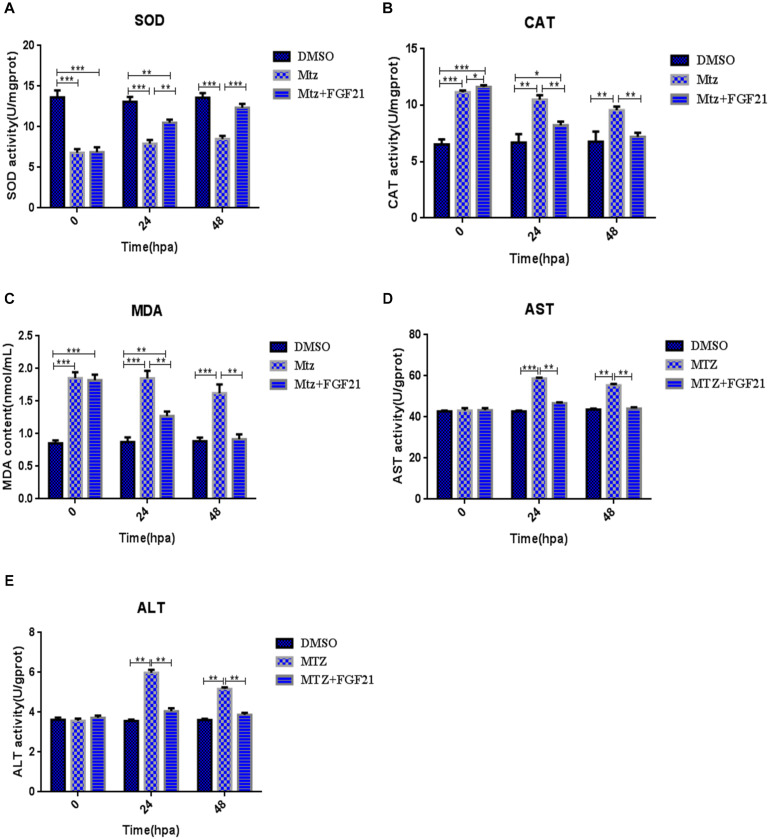
Fibroblast growth factor 21 reduces oxidative stress after liver injury. Samples were collected at 0, 24, and 48 hpa to assess oxidative stress in the damaged liver. **(A)** Evaluation of SOD activity in the control and differentially treated embryos. **(B)** Evaluation of CAT activity in the control and differentially treated embryos. **(C)** Evaluation of MDA content in the control and differentially treated embryos. **(D)** Evaluation of AST activity in the control and differentially treated embryos. **(E)** Evaluation of ALT activity in the control and differentially treated embryos. DMSO group: without Mtz treatment, DMSO was applied. Mtz group: after Mtz treatment, grow naturally. Mtz+FGF21 group: after Mtz treatment, treated with FGF21 standard protein. Significance: *p< 0.05, **p< 0.01, and ***p< 0.001 vs. DMSO group. Data are expressed as mean ± SEM (*n* = 40 per group, repeat 3 times for each test).

Oxidative stress is a key factor in liver damage, especially during the repair period after injury. Therefore, we explored whether FGF21 has a role in regulating the oxidative stress level in the regeneration of the liver after injury. The results showed that compared with the Mtz group, after 24 hpa treatment with Mtz+FGF21, FGF21 efficiently increased the level of SOD—the key enzyme against oxidative stress—in the damaged liver (Figure 3A). This effect lasted up to 48 hpa. In addition, AST, ALT, CAT, and MDA were analyzed and processed at 24 hpa and 48 hpa, respectively; the results showed that FGF21 treatment significantly reduced the activity of these enzymes in the liver tissue (Figures 3B–E). Altogether, these results indicate that FGF21 improves the antioxidant capacity of liver tissues and significantly reduces oxidative stress levels and ALT and AST activities, which may play a positive role in liver regeneration after injury.

### Fibroblast Growth Factor 21 Accelerates Liver Cell Regeneration After Injury

**FIGURE 4:**
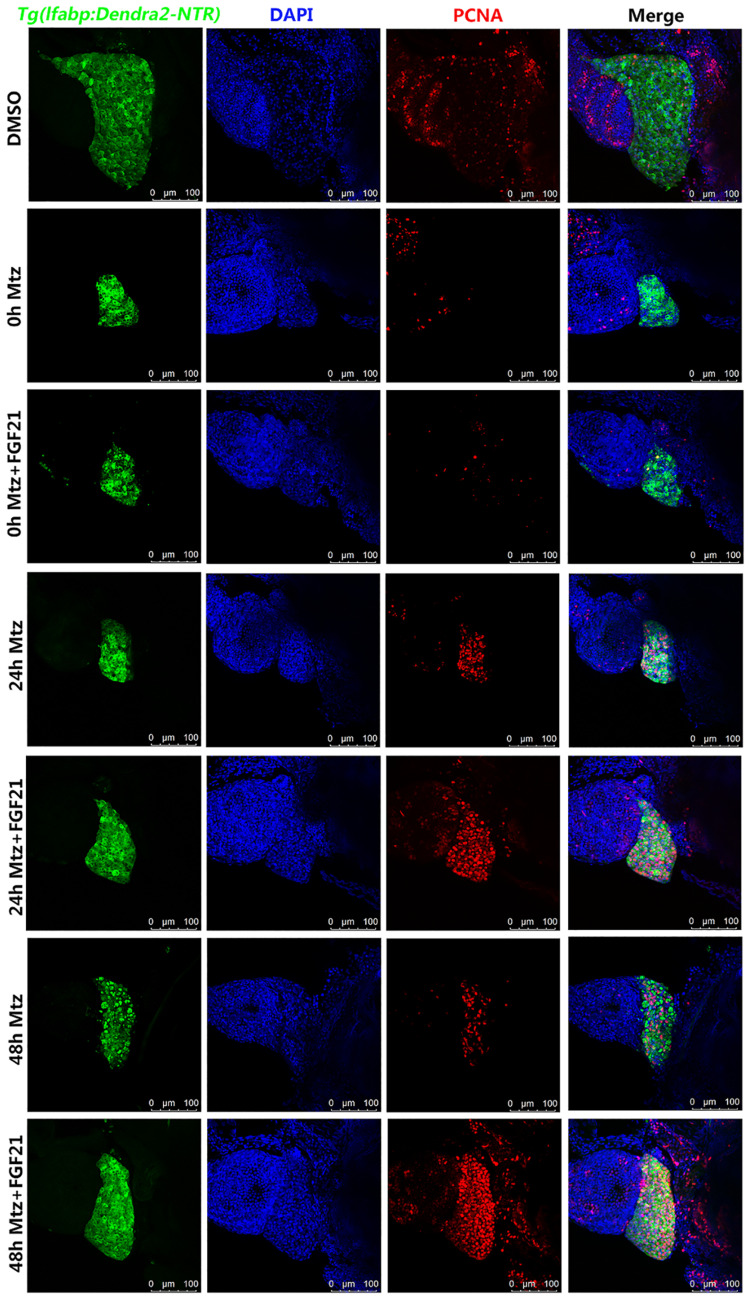
Fibroblast growth factor 21 accelerates liver cell regeneration after injury. Samples were collected at 0, 24, and 48 hpa to assess the production of new cells in the damaged liver. PCNA assay performed to assess the level of the formation of new cells in the damaged liver. Liver cells (green fluorescent Denra-NTR), stromal cells (blue fluorescent DAPI), and PCNA positive cells (red fluorescent). DMSO group: without Mtz treatment, DMSO was applied. Mtz group: after Mtz treatment, grow naturally. Mtz+FGF21 group: after Mtz treatment, treated with FGF21 standard protein (*n* = 40 per group, repeat 3 times for each test). Scale bar = 100 μm.

To investigate whether FGF21 accelerates the regeneration of liver cells after injury, we measured the levels of liver cell proliferation marker. The results showed that PCNA (red fluorescence) was expressed in liver cells (green fluorescence Denra-NTR) and stromal cells (blue fluorescence DAPI). Compared with the Mtz group, the number of PCNA-positive cells at 24 hpa and 48 hpa in the Mtz+FGF21 treatment group significantly increased (Figure 4 and Supplementary Figure 3). These results indicate that FGF21 accelerates the regeneration of liver cells after injury; so, it is a key factor in determining liver regeneration after injury.

### Fibroblast Growth Factor 21 Promotes Autophagy of Injured Liver Cells

**FIGURE 5:**
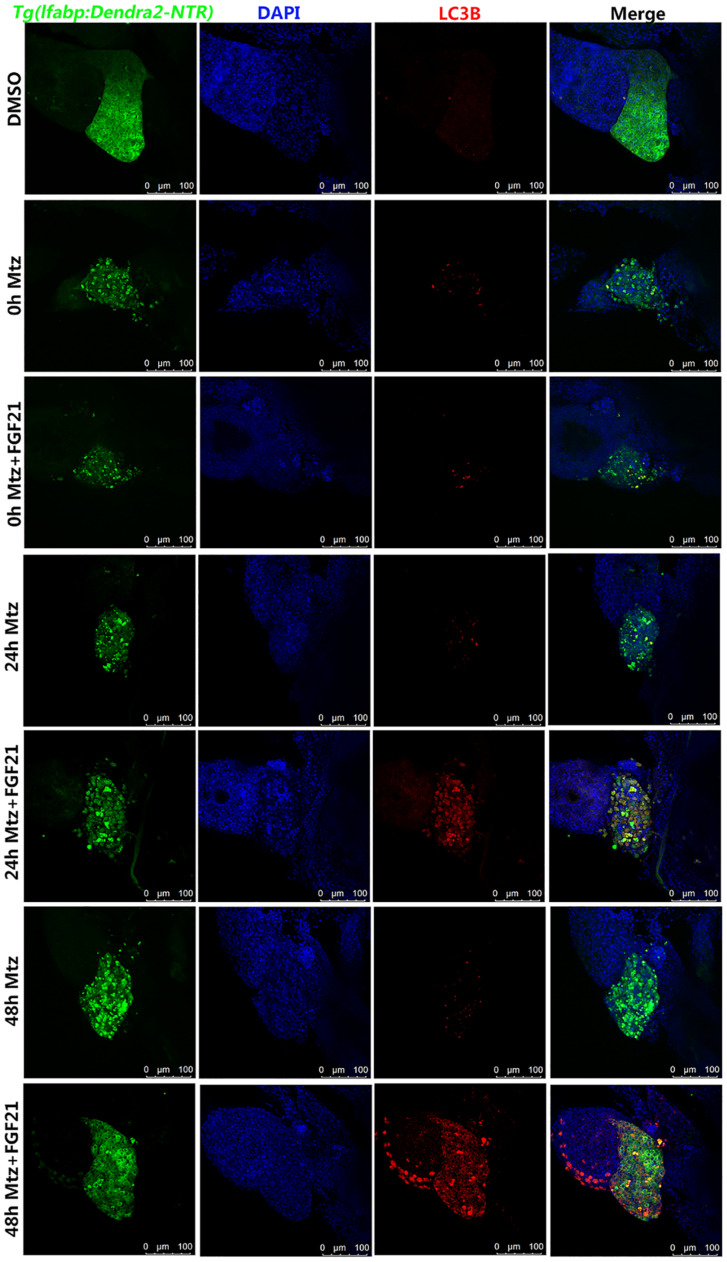
Fibroblast growth factor 21 promotes autophagy after liver injury. Samples were collected at 0, 24, and 48 hpa for the evaluation of autophagy. LC3B assay was performed to assess the level of autophagy in the damaged liver. Liver cells (green fluorescent Denra-NTR), stromal cells (blue fluorescent DAPI), and autophagy positive cells (red fluorescent LC3B). DMSO group: without Mtz treatment, DMSO was applied. Mtz group: after Mtz treatment, grow naturally. Mtz+FGF21 group: after Mtz treatment, treated with FGF21 standard protein (*n* = 40 per group, repeat 3 times for each test). Scale bar = 100 μm.

The activity of FGF21 in liver cell regeneration, apoptosis, and oxidative stress may partly stem from its role in the regulation of autophagy. Hence, to investigate whether FGF21 activates autophagy, the level of autophagy-related protein marker (LC3B) was analyzed. LC3B is an important factor required for autophagosomes. We found that compared with Mtz group, the number of cells with LC3B-labeled autophagosomes in the liver at 24 hpa and 48 hpa was higher in the Mtz+FGF21 group (Figure 5 and Supplementary Figure 4). These results indicate that FGF21 promotes autophagy in liver cells after zebrafish liver injury.

### Chloroquine Inhibits Fibroblast Growth Factor 21 Function in Liver Regeneration

In order to examine whether the increased autophagy effect of FGF21 really promotes liver regeneration, we used the well-known autophagy inhibitor CQ together with FGF21 to evaluate the results. Immunofluorescence results showed that CQ significantly reduced the number of cells with LC3B-labeled autophagosomes in the liver at 48 hpa (Figure 5 and Supplementary Figure 5). Hence, CQ successfully inhibited FGF21-stimulated autophagy in liver cells. Moreover, compared with the Mtz+FGF21 group, the 24 and 48 hpa liver regeneration rates in the Mtz+FGF21+CQ group were significantly reduced (Supplementary Figure 6). Taken together, these results indicate that CQ greatly reverses the benefits of FGF21 on liver regeneration. Therefore, it can be speculated that activation of autophagy is the main mechanism of FGF21 to promote liver regeneration.

### Chloroquine Inhibits the Effect of Fibroblast Growth Factor 21 on Oxidative Stress and Apoptosis During Liver Regeneration

Next, we assessed whether CQ co-administration affects the results of FGF21-treated liver cell regeneration. Compared with the Mtz+FGF21 group, CQ significantly increased the number of TUNEL-positive cells at 48 hpa (Figure 2A and Supplementary Figure 7A); it also promoted the proapoptotic genes *bax* and *p53* (Supplementary Figures 7B,D), which were upregulated at 24 hpa, and the antiapoptotic gene *bcl2* was downregulated at 48 hpa, indicating that FGF21-induced autophagy is the main reason for inhibited apoptosis in the injured liver (Supplementary Figure 7C). In addition, CQ significantly reduced the levels of SOD (Supplementary Figure 8A) and increased the levels of AST, ALT, MDA, and CAT at 24 hpa and 48 hpa (Supplementary Figures 8B–E). Taken together, our results indicate that FGF21 increases autophagy level, inhibits cell apoptosis, and reduces oxidative stress in the damaged liver, thereby ultimately improving the liver’s regenerative capacity.

### Fibroblast Growth Factor 21 Activates the AMPK-mTOR Signaling Pathway to Upregulate Autophagy to Accelerate Liver Regeneration

**FIGURE 6:**
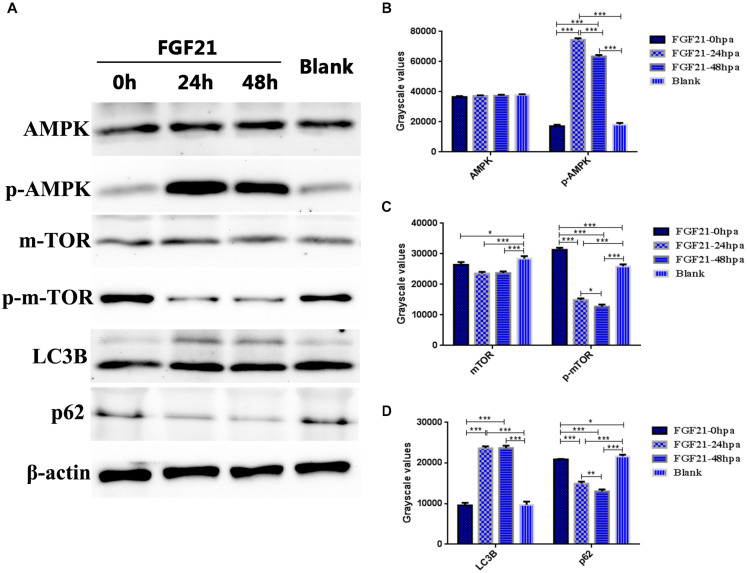
Fibroblast growth factor 21 up-regulates autophagy through AMPK-mTOR signaling pathway. Samples were collected from three groups at 0, 24, and 48 hpa. **(A)** Western blotting showing levels of AMPK, p-AMPK, mTOR, p-mTOR, LC3B, and P62, corrected for β-actin as internal control. **(B)** Quantification of immunohybridization signal of AMPK and *p*-AMPK by grayscale values. **(C)** Quantification of immunohybridization signal of mTOR and *p*-mTOR by grayscale values. **(D)** Quantification of immunohybridization signal of LC3B and P62 by grayscale values. DMSO group: without Mtz treatment, DMSO was applied. Mtz group: after Mtz treatment, grow naturally. Mtz+FGF21 group: after Mtz treatment, treated with FGF21 standard protein. Significance: *p < 0.05, **p< 0.01, and ***p< 0.001 vs. DMSO group. Data are expressed as mean ± SEM (*n* = 40 per group, repeat 3 times for each test).

The AMPK-mTOR signaling pathway plays a key role in autophagy. Thus, we evaluated the expression levels of AMPK, p-AMPK, mTOR, p-mTOR, p62, and LC3B in the damaged liver after FGF21 treatment. The results of western blot indicated that in the FGF21 treatment group, the expression of p-AMPK was upregulated at 24 hpa and 48 hpa; in contrast, the expression of p-mTOR was downregulated. The expression level of AMPK was not significantly different between the two groups (Figures 6A–C), suggesting that FGF21 triggers the AMPK-mTOR pathway. In addition, the level of LC3B in the FGF21 group was upregulated at 24 and 48 hpa, while the expression level of p62 was downregulated (Figures 6A,D). In addition, we increased the expression levels of other autophagy-related genes (*Beclin1*, *CTSD*, *VPS34*) (Supplementary Figure 9), and the expression results of these genes were similar to the expression trend of LC3B. In summary, these results confirm that FGF21 activates autophagy in liver cells through the AMPK-mTOR signaling pathway and accelerates the regeneration of damaged liver.

## Discussion

The replication capacity of liver cells and the regeneration capacity of the liver are fascinating. However, the ability of liver cells to self-regenerate is insufficient in the last stage of chronic liver disease, which is due to the loss of cell proliferation ability and the extreme loss of liver cells. The only currently available treatment is liver transplantation, but the number of donors is insufficient (Duncan et al., 2009).

In previous studies, zebrafish have been shown to be an efficient model system for studying regeneration of organs, such as kidney and liver (Gerlach and Wingert, 2013; Zhang et al., 2013; Diep et al., 2020). Larvae of zebrafish start eating 5 days after fertilization; their liver is then fully functional like in adults, and it can produce bile, store glycogen, and participate in lipid homeostasis (Chu and Sadler, 2009; Pimtong et al., 2020). Thus, it is ideal to study liver regeneration in the larvae stage after 5 dpf as they are like adult zebrafish, mature liver parenchymal cells (hepatocyte) have been developed (Choi et al., 2015). Bacterial NTR and its substrate MTZ have been shown to induce conditional targeted cell ablation in zebrafish, which can be used to study the regeneration process (Curado et al., 2007). In order to observe the process of hepatocyte regeneration more intuitively, especially in the absence of hepatocytes, a *Tg(lfabp:Dendra 2-NTR)^*cq1*^* zebrafish transgenic line was constructed to study Mtz targeting to induce NTR in the liver, causing targeted ablation of the liver in order to observe its regeneration (He et al., 2014).

In this study, Mzt was used to target the ablation of the liver but 25% of its hepatocytes were retained and FGF21 was given. The results of the present study indicate that FGF21 plays a key role in hepatocyte survival, and its exogenous administration improves the viability of liver by activating autophagy and subsequently reducing oxidative stress and inhibiting apoptosis.

Earlier, it has been shown that FGF21 can repair myocardial ischemia/reperfusion injury by promoting autophagy (Ren et al., 2019). Similarly, FGF21 has a role in the inhibition of apoptosis and oxidative stress (Opoku et al., 2020; Yu et al., 2015). Therefore, we assumed that FGF21 can reduce cell death in damaged liver, which was verified in our current work. In the process of injury and repair, when the blood supply to the damaged tissue is restored, the oxygen molecules transported in the blood can easily react to form superoxide anions. The cell membrane is destroyed by lipid peroxidation, leading to cell death and tissue necrosis. MDA and CAT are produced in the process (Halliwell and Chirico, 1993; Tsikas, 2017).

As previously reported in the literature, enhanced autophagy can inhibit oxidative stress (Filomeni et al., 2015). Therefore, we considered that FGF21 may promote survival of liver cells by preventing the accumulation of oxidative stress products. According to our results, FGF21 increases the expression of SOD, and SOD can alleviate oxidative stress and reduce the levels of MDA and CAT (Aydin, 2021). ALT and AST are commonly used serum biochemical indicators for evaluating liver function (Zhao et al., 2020); when hepatocytes are diseased or injured, the permeability of cell membranes increases, and ALT and AST are quickly released into the blood, leading to an increase in their blood levels. Therefore, after drug-induced liver injury, the serum ALT and AST levels significantly increase. In this study, after FGF21 treatment, ALT and AST levels in zebrafish were significantly reduced. In summary, these results indicate that FGF21 is an oxidative stress inhibitor in the damaged liver and can also help the liver to restore its function.

Fibroblast Growth Factor 21 can effectively inhibit cell apoptosis (Tabari et al., 2019; Wang et al., 2019). For example, it has been shown that FGF21 inhibits apoptosis by activating the PI3K/Akt signaling pathway to treat neonatal rats with hypoxic-ischemic brain injury (Ye et al., 2019). As apoptosis plays a major role in the survival of damaged liver cells, here, we explored the effect of FGF21 on apoptosis. We found that damaged livers treated with FGF21 showed reduced cell DNA damage, which may be the result of inhibited cell apoptosis. This result was also confirmed in experiments; we showed that the proapoptotic genes *bax* and *p53* in the damaged liver tissue were significantly reduced after FGF21 treatment, whereas the antiapoptotic protein *bcl2* was upregulated, which indicates that FGF21 can inhibit the level of apoptosis in the injured liver.

Autophagy is the main process of the cell’s own waste degradation, a known target pathway of FGF21, and an effective regulator of cell apoptosis and oxidative stress (Zhou et al., 2019). It can effectively maintain cell homeostasis. When tissues are damaged, autophagy can reduce the release of toxins from damaged organelles and eliminate waste on time (Levine and Klionsky, 2004). To deeply analyze the mechanism by which FGF21 accelerates liver regeneration, the role of autophagy in liver injury was studied. Here, we showed that FGF21 led to an increase in the content of autophagy markers. After autophagy was inhibited by CQ, the oxidative stress and apoptosis activities in liver tissues increased, and the survival rate of damaged liver was reduced. These results indicate that FGF21 inhibits apoptosis and oxidative stress by upregulating autophagy of damaged liver hepatocyte, thereby ultimately improving the survival of damaged liver.

It has been reported in the literature that FGF21 activates autophagy in skin flaps through the AMPK-mTOR signaling pathway to improve skin flap regeneration (Zhou et al., 2019). In this study, we proved that the AMPK-mTOR signaling pathway was activated after using FGF21 to treat damaged liver, which initiated the expression of the autophagosome-forming protein LC3B, thereby enhancing autophagy in the damaged liver. In turn, FGF21 inhibits cell apoptosis and reduces oxidative stress, which ultimately leads to increased liver cell viability. In addition, Yan et al. (2021) reported that the pathway for AMPK to activate autophagy may not only depend on mTOR, but also on the activation of SIRT1/PGC-1a, which provides us with new ideas for the study of FGF21. We will further prove this in other experiments. In short, these results provide strong evidence and underlying mechanisms for the therapeutic benefits of FGF21 on liver regeneration and highlight the clinical transformation potential of FGF21, but further validation is needed.

## Data Availability Statement

The original contributions presented in the study are included in the article/Supplementary Material, further inquiries can be directed to the corresponding author/s.

## Author Contributions

FG and XL conceived of the study and participated in its design and coordination. WQ carried out the experimental work and wrote the manuscript. TS, JG, ZJ, and DL were responsible for animal experiments and immunohistochemistry experiment. MN gave a hand for spell and grammar check. HL was responsible for analyzing the data. All authors read and approved the final manuscript.

## Conflict of Interest

The authors declare that the research was conducted in the absence of any commercial or financial relationships that could be construed as a potential conflict of interest.

## Publisher’s Note

All claims expressed in this article are solely those of the authors and do not necessarily represent those of their affiliated organizations, or those of the publisher, the editors and the reviewers. Any product that may be evaluated in this article, or claim that may be made by its manufacturer, is not guaranteed or endorsed by the publisher.

## Supplementary Material

Supplementary Figure 1 Quantification of TUNEL positive cells. TUNEL assay performed to assess the level of DNA damage in the damaged liver. Samples were collected at 0, 24, and 48 hpa. DMSO group: without Mtz treatment, DMSO was applied. Mtz group: after Mtz treatment, grow naturally. Mtz+FGF21 group: after Mtz treatment, treated with FGF21 standard protein. Significance: ^∗∗^*p*< 0.01 vs. DMSO group. Data are expressed as mean± SEM (*n*= 40 per group, repeat 3 times for each test).

Supplementary Figure 2 Protein expression levels of apoptotic markers (p53, bcl2, and bax). Samples were collected at 0, 24, and 48 hpa. **(A)** Western blotting showing levels of p53, bcl2, and bax, corrected for β-actin as internal control. **(B)** Quantification of immunohybridization signal by grayscale values. Significance: ^∗^*p*< 0.05 and ^∗∗∗^*p*< 0.001 vs. Blank group. Data are expressed as mean± SEM (*n*= 40 per group, repeat 3 times for each test).

Supplementary Figure 3 Quantification of PCNA positive cells. Samples were collected at 0, 24, and 48 hpa to assess the production of new cells in the damaged liver. PCNA assay performed to assess the level of the formation of new cells in the damaged liver. DMSO group: without Mtz treatment, DMSO was applied. Mtz group: after Mtz treatment, grow naturally. Mtz+FGF21 group: after Mtz treatment, treated with FGF21 standard protein. Significance: ^∗^*p*< 0.05 and ^∗∗^*p*< 0.01 vs. DMSO group. Data are expressed as mean ± SEM (*n*= 40 per group, repeat 3 times for each test).

Supplementary Figure 4 Quantification of LC3B positive cells. Samples were collected at 0, 24, and 48 hpa for the evaluation of autophagy. LC3B assay was performed to assess the level of autophagy in the damaged liver. DMSO group: without Mtz treatment, DMSO was applied. Mtz group: after Mtz treatment, grow naturally. Mtz+FGF21 group: after Mtz treatment, treated with FGF21 standard protein. Significance: ^∗^*p*< 0.05 and ^∗∗∗^*p*< 0.001 vs. DMSO group. Data are expressed as mean ± SEM (*n*= 40 per group, repeat 3 times for each test).

Supplementary Figure 5 CQ inhibits FGF21-stimulated autophagy in liver cells. Samples were collected at 48 hpa for the evaluation of autophagy. LC3B assay was performed to assess the level of autophagy in the damaged liver. Liver cells (green fluorescent Denra-NTR), stromal cells (blue fluorescent DAPI), and autophagy positive cells (red fluorescent LC3B). Mtz+FGF21+CQ group: after treatment with Mtz, treated with FGF21 standard protein and CQ at the same time. Scale bar = 100 μm (*n*= 40 per group, repeat 3 times for each test).

Supplementary Figure 6 CQ inhibits the promoting effect of FGF21 on liver regeneration. After establishing the liver targeted ablation model, FGF21 and FGF21+CQ were administered and the treatment effects were evaluated. Liver regeneration at several time points after FGF21 and FGF21+CQ treatment of the targeted ablated liver. DMSO group: without Mta treatment, DMSO was applied. Mtz+FGF21 group: after Mtz treatment, treated with FGF21 standard protein. Mtz+FGF21+CQ group: After treatment with Mtz, treated with FGF21 standard protein and CQ at the same time. Scale bar = 100 μm (*n*= 40 per group, repeat 3 times for each test).

Supplementary Figure 7 CQ inhibits the effect of FGF21 on apoptosis during liver regeneration. **(A)** TUNEL assay was performed to assess the level of DNA damage in the Mtz+FGF21+CQ group (nuclei, blue; damaged DNA, red; liver cells, green). **(B)** RT-qPCR detection of bax mRNA of four groups at 0, 24, and 48 hpa. **(C)** RT-qPCR detection of bcl2 mRNA of four groups at 0, 24, and 48 hpa. **(D)** RT-qPCR detection of p53 mRNA of four groups at 0, 24, and 48 hpa. DMSO group: without Mta treatment, DMSO was applied. Mtz group: after Mtz treatment, grow naturally. Mtz+FGF21 group: after Mtz treatment, treated with FGF21 standard protein. Mtz+FGF21+CQ group: after treatment with Mtz, treated with FGF21 standard protein and CQ at the same time. Significance: ^∗^*p*< 0.05, ^∗∗^*p*< 0.01, and ^∗∗∗^*p*< 0.001 vs. DMSO group. Data are expressed as mean ± SEM (*n* = 40 per group, repeat 3 times for each test). Scale bar = 100 μm.

Supplementary Figure 8 CQ inhibits the effect of FGF21 on oxidative stress during liver regeneration. Samples were collected at 0, 24, and 48 hpa to assess oxidative stress in the damaged liver. **(A)** Evaluation of SOD activity in the control and differentially treated embryos. **(B)** Evaluation of CAT activity in the control and differentially treated embryos. **(C)** Evaluation of MDA content in the control and differentially treated embryos. **(D)** Evaluation of AST activity in the control and differentially treated embryos. **(E)** Evaluation of ALT activity in the control and differentially treated embryos. DMSO group: without Mta treatment, DMSO was applied. Mtz group: after Mtz treatment, grow naturally. Mtz+FGF21 group: after Mtz treatment, treated with FGF21 standard protein. Mtz+FGF21+CQ group: after treatment with Mtz, treated with FGF21 standard protein and CQ at the same time. Significance: ^∗^*p*< 0.05, ^∗∗^*p*< 0.01, and ^∗∗∗^*p*< 0.001 vs. DMSO group. Data are expressed as mean ± SEM (*n* = 40 per group, repeat 3 times for each test).

Supplementary Figure 9 Expression levels of autophagy-related genes (Beclin1, CTSD, and VPS34). **(A)** RT-qPCR detection of *Beclin1* mRNA at 0, 24, and 48 hpa. **(B)** RT-qPCR detection of *CTSD* mRNA at 0, 24, and 48 hpa. **(C)** RT-qPCR detection of *VPS34* mRNA at 0, 24, and 48 hpa. Significance: ^∗^*p*< 0.05 and ^∗∗^*p*< 0.01 vs. Blank group. Data are expressed as mean ± SEM (*n*= 40 per group, repeat 3 times for each test).

Supplementary Table 1 The effect of different concentrations of FGF21 on zebrafish liver regeneration. FGF21 (1 mg/mL) was prepared with zebrafish rearing water and diluted gradually to the final concentration. According to the results of preliminary experiments, the rescue phenotype of zebrafish juveniles after liver injury is not obvious when the drug concentration is below 100 ng/mL, and the mortality rate is relatively high. When the concentration is greater than 200 ng/mL, there are almost no dead juveniles. Therefore, the concentration of the test solution was set at 200 ng/mL. The experiment was repeated three times.

Supplementary Table 2 qRT-PCR primer sequences.
