# Supplementary material for: Fibroblast Growth Factor 21 Augments Autophagy and Reduces Apoptosis in Damaged Liver to Improve Tissue Regeneration in Zebrafish
Source: Front Cell Dev Biol. 2021 Oct 22;9:756743. doi: 10.3389/fcell.2021.756743 (PMC8570170; doi:10.3389/fcell.2021.756743)
Supplement: Supplementary Figure 1 — Quantification of TUNEL positive cells. TUNEL assay performed to assess the level of DNA damage in the damaged liver. Samples were collected at 0, 24, and 48 hpa. DMSO group: without Mtz treatment, DMSO was applied. Mtz group: after Mtz treatment, grow naturally. Mtz+FGF21 group: after Mtz treatment, treated with FGF21 standard protein. Significance: ∗∗p< 0.01 vs. DMSO group. Data are expressed as mean± SEM (n= 40 per group, repeat 3 times for each test). [file Data_Sheet_1.docx]

Supplementary Material

# Supplementary Figures and Tables

## Supplementary Figures


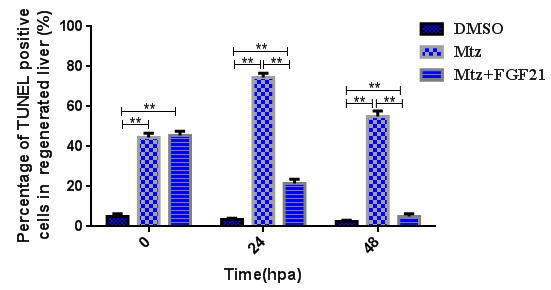


**Supplementary Figure 1. Quantification of TUNEL positive cells.** TUNEL assay performed to assess the level of DNA damage in the damaged liver. Samples were collected at 0, 24, and 48 hpa. DMSO group: without Mtz treatment, DMSO was applied. Mtz group: after Mtz treatment, grow naturally. Mtz+FGF21 group: after Mtz treatment, treated with FGF21 standard protein. Significance: ***p*< 0.01 vs. DMSO group. Data are expressed as mean ± SEM (n = 40 per group, repeat 3 times for each test).


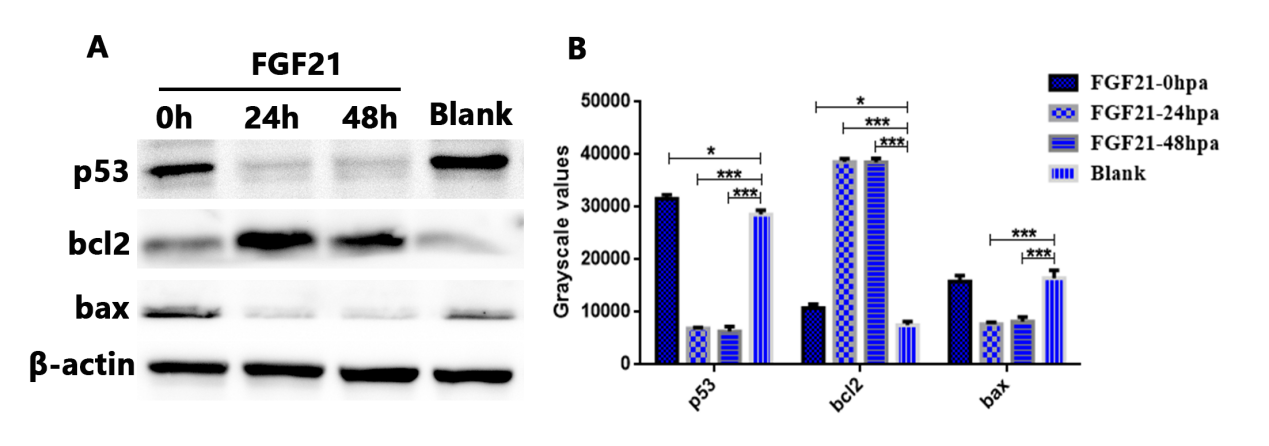


**Supplementary Figure 2. Protein expression levels of apoptotic markers(p53, bcl2, and bax).** Samples were collected at 0, 24, and 48 hpa. (A) Western blotting showing levels of p53, bcl2, and bax, corrected for β-actin as internal control. (B) Quantification of immunohybridization signal by grayscale values. Significance: **p* < 0.05 and ****p* < 0.001 vs. Blank group. Data are expressed as mean ± SEM (n = 40 per group, repeat 3 times for each test).


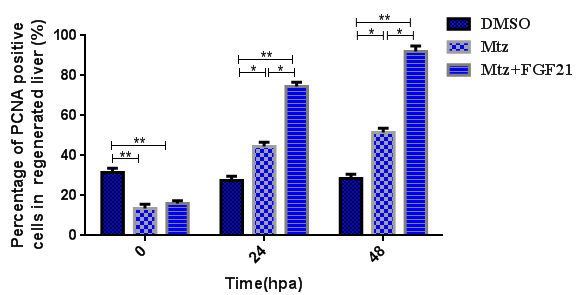


**Supplementary Figure 3. Quantification of PCNA positive cells.** Samples were collected at 0, 24, and 48 hpa to assess the production of new cells in the damaged liver. PCNA assay performed to assess the level of the formation of new cells in the damaged liver. DMSO group: without Mtz treatment, DMSO was applied. Mtz group: after Mtz treatment, grow naturally. Mtz+FGF21 group: after Mtz treatment, treated with FGF21 standard protein. Significance: **p* < 0.05 and ***p*< 0.01 vs. DMSO group. Data are expressed as mean ± SEM (n = 40 per group, repeat 3 times for each test).


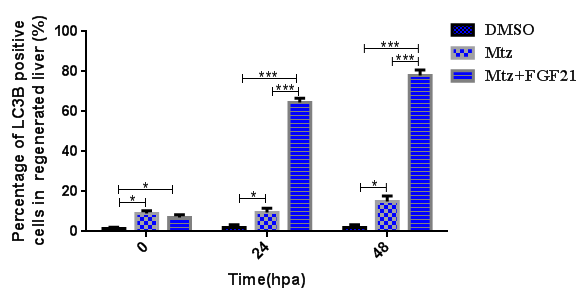


**Supplementary Figure 4. Quantification of LC3B positive cells.** Samples were collected at 0, 24, and 48 hpa for the evaluation of autophagy. LC3B assay was performed to assess the level of autophagy in the damaged liver. DMSO group: without Mtz treatment, DMSO was applied. Mtz group: after Mtz treatment, grow naturally. Mtz+FGF21 group: after Mtz treatment, treated with FGF21 standard protein. Significance: **p* < 0.05 and ****p* < 0.001 vs. DMSO group. Data are expressed as mean ± SEM (n = 40 per group, repeat 3 times for each test).


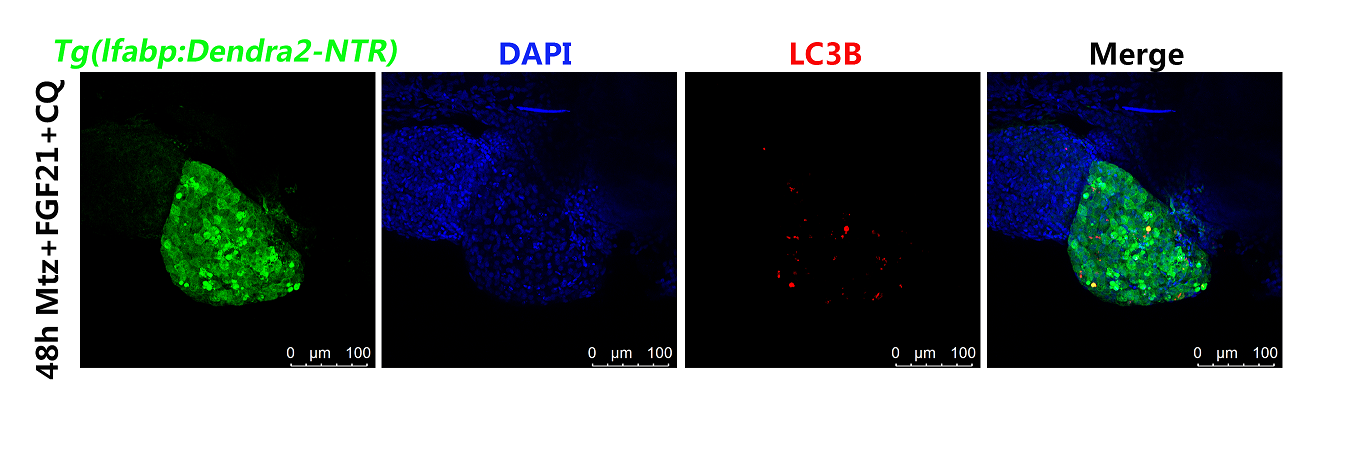


**Supplementary Figure 5.** **CQ inhibits FGF21-stimulated autophagy in liver cells.** Samples were collected at 48 hpa for the evaluation of autophagy. LC3B assay was performed to assess the level of autophagy in the damaged liver. Liver cells (green fluorescent Denra-NTR), stromal cells (blue fluorescent DAPI), and autophagy positive cells (red fluorescent LC3B). Mtz+FGF21+CQ group: after treatment with Mtz, treated with FGF21 standard protein and CQ at the same time. Scale bar, 100 µm (n = 40 per group, repeat 3 times for each test).


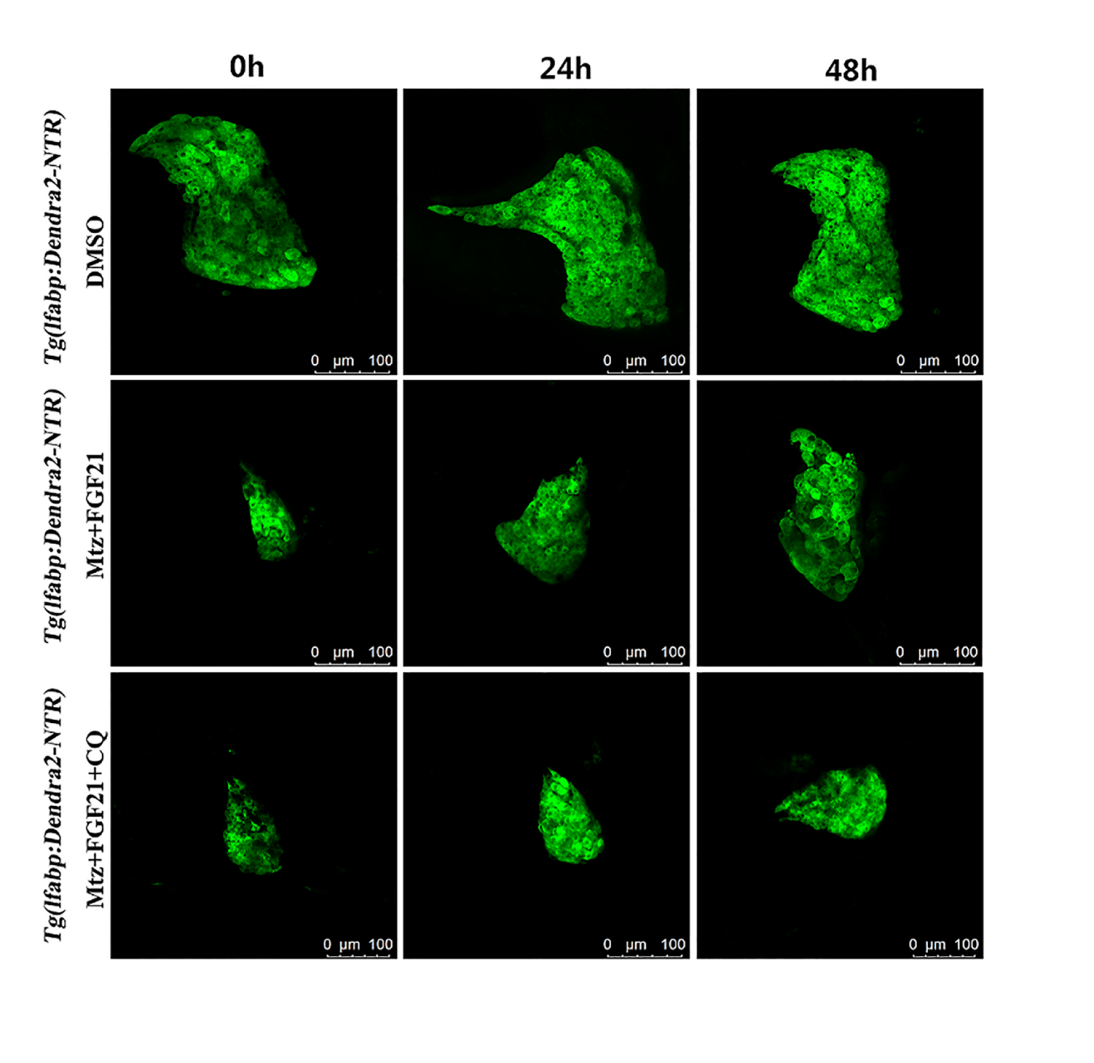


**Supplementary Figure 6. CQ inhibits the promoting effect of FGF21 on liver regeneration.** After establishing the liver targeted ablation model, FGF21 and FGF21+CQ were administered and the treatment effects were evaluated. Liver regeneration at several time points after FGF21 and FGF21+CQ treatment of the targeted ablated liver. DMSO group: without Mta treatment, DMSO was applied. Mtz+FGF21 group: after Mtz treatment, treated with FGF21 standard protein. Mtz+FGF21+CQ group: After treatment with Mtz, treated with FGF21 standard protein and CQ at the same time. Scale bar, 100 µm (n = 40 per group, repeat 3 times for each test).


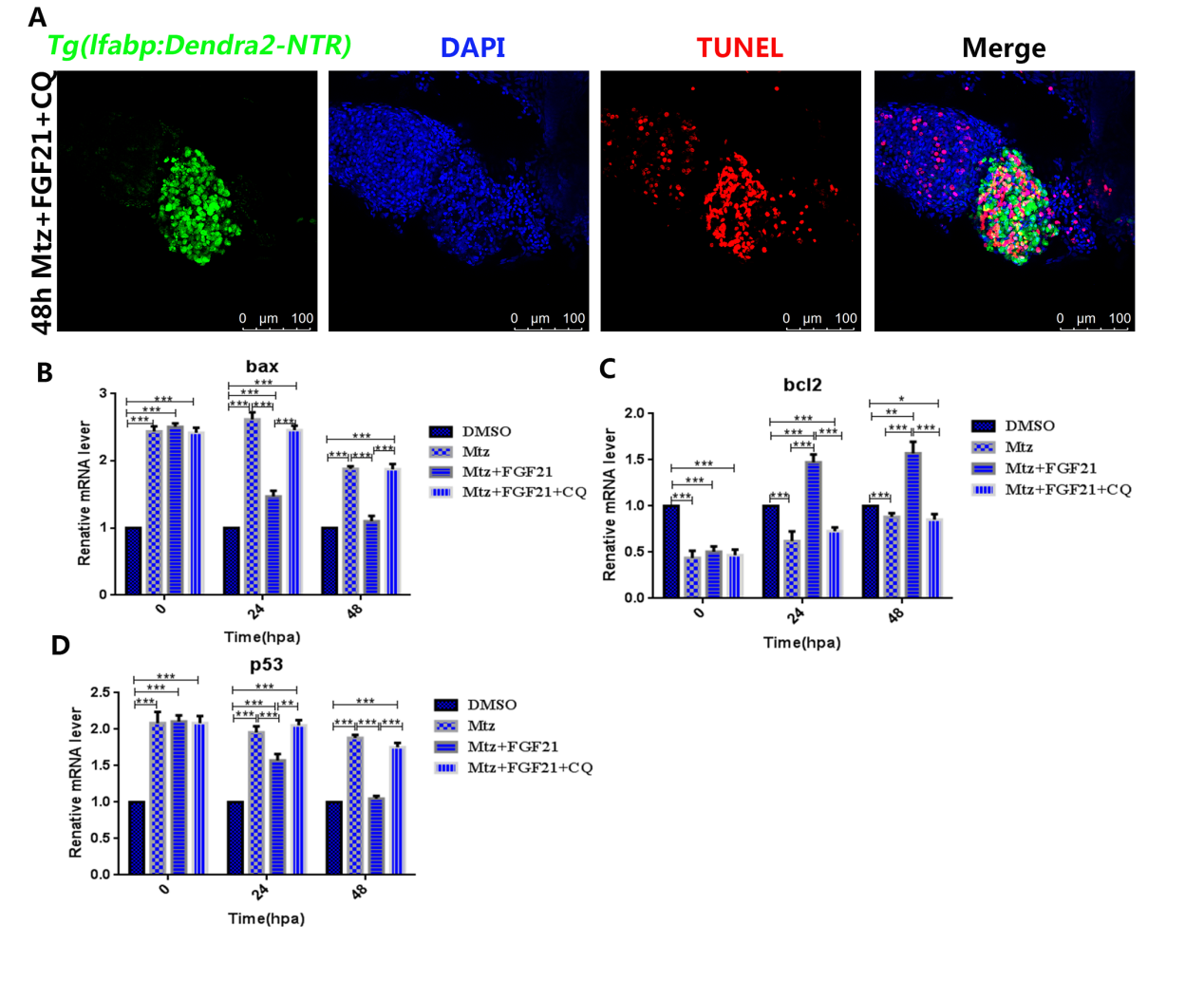


**Supplementary Figure 7. CQ inhibits the effect of FGF21 on apoptosis during liver regeneration.** (A) TUNEL assay was performed to assess the level of DNA damage in the Mtz+FGF21+CQ group (nuclei, blue; damaged DNA, red; liver cells, green). (B) RT-qPCR detection of bax mRNA of four groups at 0, 24, and 48 hpa. (C) RT-qPCR detection of bcl2 mRNA of four groups at 0, 24, and 48 hpa. (D) RT-qPCR detection of p53 mRNA of four groups at 0, 24, and 48 hpa. DMSO group: without Mta treatment, DMSO was applied. Mtz group: after Mtz treatment, grow naturally. Mtz+FGF21 group: after Mtz treatment, treated with FGF21 standard protein. Mtz+FGF21+CQ group: after treatment with Mtz, treated with FGF21 standard protein and CQ at the same time. Significance: **p* < 0.05, ***p* < 0.01, and ****p* < 0.001 vs. DMSO group. Data are expressed as mean ± SEM (n = 40 per group, repeat 3 times for each test). Scale bar, 100 µm.


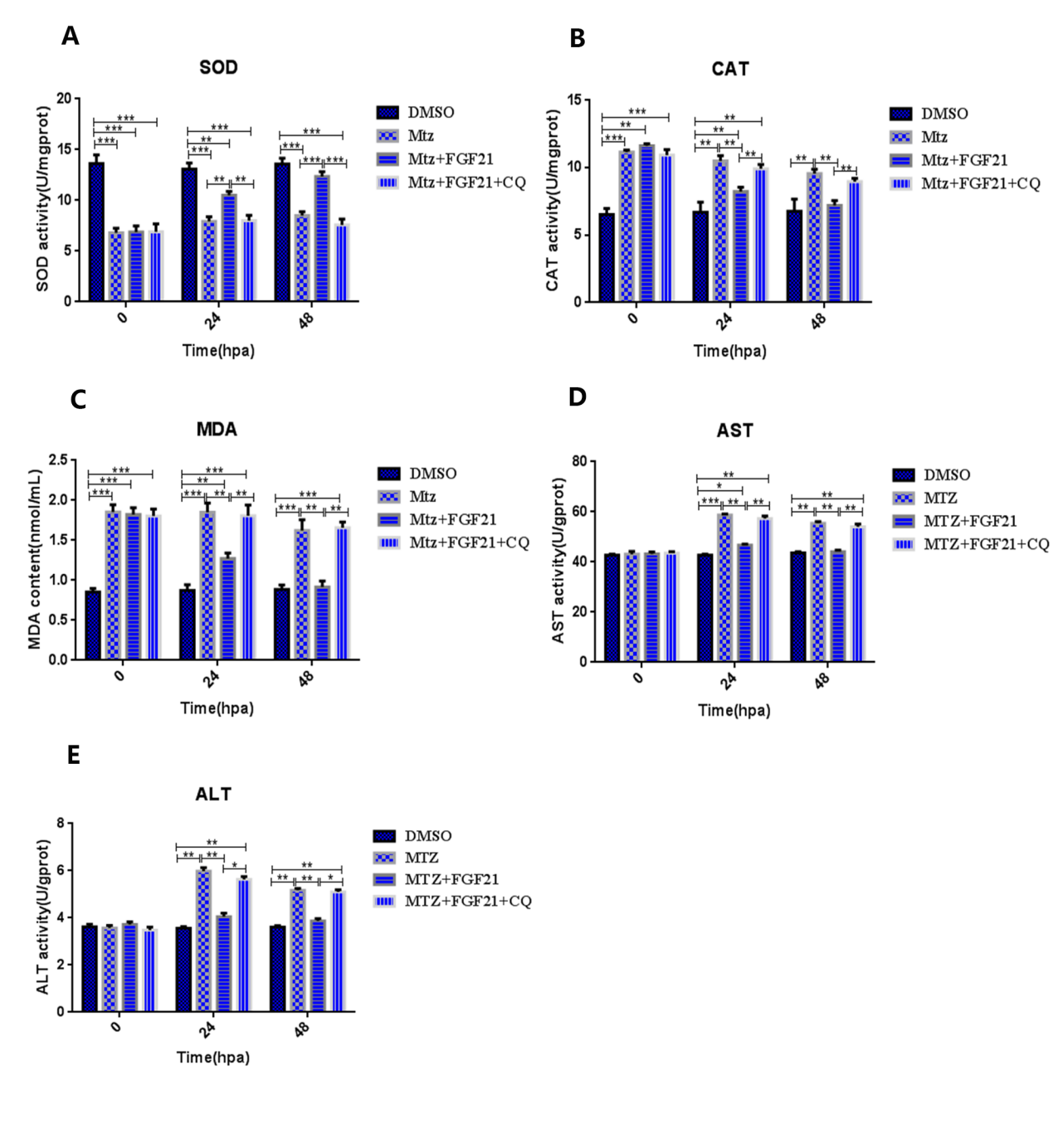


**Supplementary Figure 8. CQ inhibits the effect of FGF21 on oxidative stress during liver regeneration.** Samples were collected at 0, 24, and 48 hpa to assess oxidative stress in the damaged liver. (A) Evaluation of SOD activity in the control and differentially treated embryos. (B) Evaluation of CAT activity in the control and differentially treated embryos. (C) Evaluation of MDA content in the control and differentially treated embryos. (D) Evaluation of AST activity in the control and differentially treated embryos. (E) Evaluation of ALT activity in the control and differentially treated embryos. DMSO group: without Mta treatment, DMSO was applied. Mtz group: after Mtz treatment, grow naturally. Mtz+FGF21 group: after Mtz treatment, treated with FGF21 standard protein. Mtz+FGF21+CQ group: after treatment with Mtz, treated with FGF21 standard protein and CQ at the same time. Significance: **p* < 0.05, ***p* < 0.01, and ****p* < 0.001 vs. DMSO group. Data are expressed as mean ± SEM (n = 40 per group, repeat 3 times for each test).


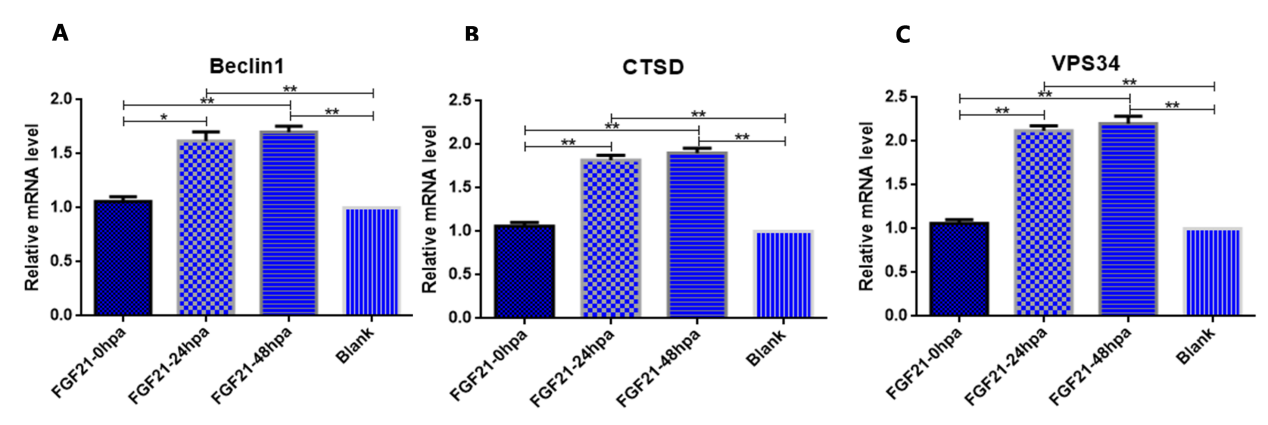


**Supplementary Figure 9. Expression levels of autophagy-related genes (Beclin1, CTSD, VPS34).** (A) RT-qPCR detection of *Beclin1* mRNA at 0, 24, and 48 hpa. (B) RT-qPCR detection of *CTSD* mRNA at 0, 24, and 48 hpa. (C) RT-qPCR detection of *VPS34* mRNA at 0, 24, and 48 hpa. Significance: **p* < 0.05 and ***p*< 0.01 vs. Blank group. Data are expressed as mean ± SEM (n = 40 per group, repeat 3 times for each test).

**Supplementary Table 1.** The effect of different concentrations of FGF21 on zebrafish liver regeneration.

| Dosing concentration | 50 ng/ml | 100 ng/ml | 200 ng/ml | 400 ng/ml |
| --- | --- | --- | --- | --- |
| Mortality rate | 82.5% | 65% | 5% | 7.5% |
| Rescue rate | 17.5% | 35% | 95% | 92.5% |

FGF21 (1 mg/mL) was prepared with zebrafish rearing water and diluted gradually to the final concentration. According to the results of preliminary experiments, the rescue phenotype of zebrafish juveniles after liver injury is not obvious when the drug concentration is below 100 ng/mL, and the mortality rate is relatively high. When the concentration is greater than 200 ng/mL, there are almost no dead juveniles. Therefore, the concentration of the test solution was set at 200 ng/mL.The experiment was repeated three times.

**Supplementary Table 2.** qRT-PCR primer sequences

| bax q-PCR FP | TCGAACATGATCTGTGTCATC |
| --- | --- |
| bax q-PCR RP | TATGGCTGGGGTCACTTTTCTC |
| bcl-2 q-PCR FP | TGGCGTCCCAGGTAGATAAT |
| bcl-2 q-PCR RP | ACCGTACATCTCCACGAAGG |
| p53 q-PCR FP | CCCGGATGGAGATAACTTG |
| p53 q-PCR RP | CACAGTTGTCCATTCAGCAC |
| Beclin1 q-PCR FP | ATTGTTCAGGTGGTCTGCGT |
| Beclin1 q-PCR RP | TGAAACCCAGGCTAAACCCC |
| CTSD q-PCR FP | GAGCTGCTACTGGGAGGAAC |
| CTSD q-PCR RP | AGCGTCGGCACTTTCTTACA |
| VPS34 q-PCR FP | CCTGGCCAAGCTAACAAAAGC |
| VPS34 q-PCR RP | AACTGGTCGGCAAAGGAGAG |
| β-actin q-PCR FP | AGCACGGTATTGTGACTAACTG |
| β-actin q-PCR RP | TCGAACATGATCTGTGTCATC |
| GAPDH q-PCR FP | CGACTCCACCCATGGAAAGT |
| GAPDH q-PCR RP | CACCCTTAATGTGAGCAGAAGC |
